# 

**Published:** 1892-06-11

**Authors:** 


**Notices of Births, Deaths, and Marriages,** are inserted in this column at a charge of 2s. 6d. for an announcement not exceeding four lines.

### NOTICE TO CORRESPONDENTS.

All MS., Letters, Books for Review, and other matters intended for the Editor should be addressed The Editor, The Lodge, Porchester Square, London, W.

The Editor cannot undertake to return rejected M.S., even when accompanied by stamped directed envelope.

**Notice to Advertisers and Subscribers.**

All Advertisements, Orders for Copies of The Hospital, and Business Communications should be addressed to The Publisher, *not to the Editor*, The Hospital, 140, Strand, W.C.

The Cost of Subscription, post free, is as follows:—Per week, 2½d.; per quarter, 2s. 6d.; per half-year, 5s.; per annum, 8s. 6d. Foreign:—Per annum, 10s. 8d.; payable, in all cases, in advance.

**"Burdett's Hospital Annual," 1891—1892.**

Third year of publication. 600 pp. Boards, gilt lettering. A most complete guide to British and Colonial Hospitals, Dispensaries, Nursing and Convalescent Institutions. and Asylums. Price 3s. 6d. Published by The Scientific Press (Limited), 140, Strand, W.C.

**NOTICE.—The Index for Vol. XI. (ending March, 1892) is on sale at the Office of this Journal, price 2½d., post free.**

### The Student of Medicine.

[All communications for this Supplement should be addressed to The Editor of The Hospital, at 140, Strand, with the words, "Students' Column," written in the left hand top corner of the envelope.]

### APPOINTMENTS.

Andrew, J. Grant, M.B., C.M.Glasg., appointed Surgeon to the Dispensary of the Victoria Infirmary, Glasgow. Banks, A., L.R.C.P., M.R.C.S., appointed Assistant House Surgeon to St. Thomas's Hospital. Board, E. C., L.R.C.P.Lond., M.R.C.S., appointed Honorary Consulting Surgeon to the Bristol Royal Infirmary. Bowring, W. A., L.R.C.P., M.R.C.S., appointed Resident House Physician to St. Thomas's Hospital. Box, C. R., B.Sc.Lond., L.R.C.P., M.R.C.S., reappointed House Surgeon to St. Thomas's Hospital. Cooper, A. Tanner, M.R.C.S., L.R.C.P., appointed House Physician to the Hospital for Women, Soho Square. Cooper, H. J., M.A., M.B., B.C.Cantab, L.R.C.P., M.R.C.S., appointed Clinical Assistant in the Ear Department of St. Thomas's Hospital. Dalzell, A., L.R.C.P., M.R.C.S., reappointed Clinical Assistant in the Throat Department of St. Thomas's Hospital. Dorman, M. R. P., M.A., M.B., B.C.Cantab., L.R.C.P., M.R.C.S., appointed Clinical Assistant in the Throat Department of St. Thomas's Hospital. Duncan, J. A. H., M.B., C.M., Clinical Assistant Dundee Royal Asylum, appointed Resident Medical Officer Dundee Royal Infirmary. Fisher, J. H., L.R.C.P., M.R.C.S., appointed Assistant House Surgeon to St. Thomas's Hospital. Fisher, J., B.A., M.B., B.C.Cantab., reappointed Ophthalmic House Surgeon to St. Thomas's Hospital. Fooks, W. P., M.A., M.B., B.C.Cantab., L.R.C.P., M.R.C.S., appointed Non-Resident House Physician to St. Thomas's Hospital. Forde, T. A. M., L.R.C.P., M.R.C.S., reappointed House Surgeon to St. Thomas's Hospital. Greig, David M., M.B., F.R.C.S.Edin., appointed Assistant Surgeon to the Dundee Royal Infirmary. Gunn, Donald, F.R.C.S.Eng., L.R.C.P.Lond., appointed Ophthalmic Surgeon to the Seamen's Hospital, Greenwich. Hamilton, James, M.B., C.M.Glas., appointed Physician to the Dispensary of the Victoria Infirmary, Glasgow. Harris, Thomas, M.D.Lond., M.R.C.P., appointed Physician to the Manchester Royal Infirmary. Jenkins, Thomas Wilson, LL.D., L.R.C.P. Edin., L.F.P.S.Glas., appointed Physician to the Dispensary of the Victoria Infirmary, Glasgow. Kelloch, T. H., M.A., M.B., B.C.Cantab., F.R.C.S., L.R.C.P., reappointed House Surgeon to St. Thomas's Hospital. Kennedy, M. A., B.Sc., M.B., C.M., appointed Surgeon to the Dispensary of the Victoria Infirmary, Glasgow. Latter, C., M.A., M.B., B.C.Cantab., appointed Obstetric House Physician to St. Thomas's Hospital. Law, R. R., B.A., M.B., B.C.Cantab., appointed Clinical Assistant in the Skin Department of St. Thomas's Hospital. Lovell, C. P., M.A., M.B., B.Ch.Oxon., L.R.C.P., M.R.C.S., L.S.A., reappointed Clinical Assistant in the Skin Department of St. Thomas's Hospital. Milton, W. F. E., L.R.C.P., M.R.C.S., reappointed House Surgeon to St. Thomas's Hospital. Penberthy, Wm., M.R.C.S., L.R.C.P., appointed Assistant Medical Officer to the Nottingham Borough Asylum. Simpson, H., B.A., M.B., B.C.Cantab., L.R.C.P., M.R.C.S., appointed Clinical Assistant in the Ear Department of St. Thomas's Hospital. Stephens, Richard J., M.R.C.S., L.S.A., appointed Assistant Medical Officer to the County Asylum, Whittingham, Lancashire. Toller, S. G., L.R.C.P., M.R.C.S., reappointed Ophthalmic House Surgeon to St. Thomas's Hospital. Wainwright, W., L.R.C.P., M.R.C.S., appointed Obstetric House Physician to St. Thomas's Hospital. Wallace, F. G., M.A., M.B., B.C.Cantab., L.R.C.P., M.R.C.S., appointed Non-Resident House Physician to St. Thomas's Hospital. Walker, Norman, M.D., F.R.C.P.Edin., appointed Assistant Physician (in Dermatology) to the Edinburgh Royal Infirmary. Wright J. Crossley, M.B., B.C.Camb., appointed Medical Officer to Halifax Infirmary.

### VACANCIES.

### Resident Medical Officers.

The following vacancies are announced this week, the amount of salary in each case being given after the name of the institution:

Clayton Hospital and Wakefield Dispensary, Wakefield. Junior House Surgeon, unmarried—Honorarium, £40 per annum, with board, lodging, and washing.

Derby Borough Asylum, Rowditch, Derby, Assistant Medical Officer—Salary, £100 per annum, with board and washing.

### THE STUDENT OF MEDICINE—(*continued*).

Manchester and Salford Provident Dispensaries' Association, vacancy on the Medical Staff of the Asso[illegible]

Royal Free Hospital, Gray's Inn Road, Junior Resident Medical Officer, doubly qualified.—Board, &c.

West London Hospital, Hammersmith, W., House Physician—Board, &c.

West London Hospital, Hammersmith, W., House Surgeon—Board, &c.

Worcester General Infirmary, Assistant to House Surgeon; qualified, unmarried; to act also as Dispenser—Salary £70 per annum, with board, &c.

### Non-Resident Medical Officers.

Dental Hospital of London, Leicester Square, W.C., Dental Surgeon.
Dental Hospital of London and London School of Dental Surgery, Leicester Square, Demonstrator—Honorarium £50 per annum.
Dundee Royal Lunatic Asylum, Clinical Assistant.
Manchester Hospital for Consumption and Diseases of the Throat and Chest, Hardman Road, Deansgate, Manchester, Honorary Assistant Physician.
Mason College, Birmingham, with Queen's Faculty of Medicine, Professorship of Medicine, Lectureship on Ophthalmology, Lectureship on Dental Surgery and Dental Pathology.
North Staffordshire Infirmary and Eye Hospital, Hartshill, Stoke-on-Trent, Assistant Ophthalmic Surgeon.
Wolverhampton and District Hospital for Women, two Honorary Acting Gynæcological Surgeons.

### ATHLETICS.

### Guy's Hospital Athletic Sports.

Pleasant weather was associated with the annual sports of the above hospital, decided on the L.A.C. Ground, at Stamford Bridge last week. There was a good assemblage of spectators present, and some excellent form was witnessed during the afternoon. H. A. Munro, starting from scratch, won the mile in 4 min. 30 2-5th sec., and later on romped home first in fine style for the three miles handicap. In the Strangers' Half-mile Handicap Harold Wade distinguished himself, winning a fine race in 2 min. 1-5th sec. As was the case with Munro, the wind was decidedly against him. The officials were: Starter, Mr. F. W. Parker (this gentleman also allotting the start for the open half); judges, Messrs. A. D. Fripp and L. A. Dunn; timekeeper, Nat Perry.

100 Yards Handicap.—Heat 1: H. T. Bell, scratch, 1; H. W. Dudgeon, 7 yards start, 2; H. A. Munro, 8, 3. Won by a yard; time, 10 4-5th sec. Heat 2: H. J. M. Smith, 6 yards start, 1; F. H. Simpson, 6, 2; H. T. Hinton, 4, 3. Won by a yard and a half; time, 11 2-5th sec. Heat 3: W. P. Bush, 10 yards start, 1; L. B. Carpenter, 6, 2; C. M. Beadnell, 6, 3. Won by half a yard; time, 10 4-5th sec. Heat 4: N. V. Lower, 11 yards start, 1; S. A. Razzak, 6, 2. Won by half a yard; time, 11 sec. Final heat: Bell, 1; Bush, 2; Lower, 3; Smith, 0. Won by a yard; a moderate third; time, 10 2-5th sec.

Half-mile Handicap.—M. E. Tressider, 45 yards start, 1; H. W. Dudgeon, 35, 2; J. W. Eastment, 40, 3. Won easily; time, 2 min. 1 1-5th sec.; five ran.

Throwing the Cricket Ball.—E. Reid, 96 yards 2 ft., 1; J. H. Bettington, 93 yards 2 ft. 9 in., 2.

120 Yards Hurdle Handicap.—H. T. Bell, 5 yards start, 1; C. M. Beadnell, scratch, 2; P. R. Lawes, owes 20, 0. An easy win; time 2[illegible] 1-5th sec.

One Mile Handicap.—A. E. Maturin, 240 yards start, 1; H. A. Munro, scratch, 2; H. C. Holden, 160, 3; P. Willmott, 110, 0; E. Picnot, 160, 0; W. A. Carden, 200, 0; P. F. Henry, 240, 0. Maturin kept in front throughout, and won easily; time, 4 min. 21 sec. Munro's time was 4 min. 30 2-4th sec.

220 Yards Handicap.—H. T. Bell, scratch, 1; F. P. Bush, 18 yards start, 2; C. E. Hibbard, 13, 3; E. Reid, 10, 0; G. N. Mottram, 11, 0. Won by four yards; rather more between second and third; time, 25 2-5th sec.

Throwing the Hammer.—P. R. Lowe, 68 ft. 9 in., 1; E. N. Scott, 62 ft., 2.

Quarter-mile Handicap.—H. W. Dudgeon, 7 yards start, 1; L. B. Carpenter, 20, 2; H. S. Desprez, scratch, 3; C. E. Hibbard, 6, 0; H. C. Holden, 10, 0; S. A. Razzak, 15, 0; H. T. Hinton, 20, 0; C. M. Bedwell, 12, 0. Won by a yard; ten yards between second and third; time, 55 4-5th sec.

High Jump Handicap.—F. T. Bush, scratch, 5 ft. 6 in., 1; H. T. Bell, owes 6 in., 5 ft. 2 in., 2.

Putting the Shot.—W. D. Macdonald, owes 1 ft., 31 ft. 7½ in., 1; E. N. Scott, scratch, 30 ft. 7 in. 2; R. S. Rowland, 30 ft. 6 in., 3.

880 Yards Strangers' Handicap.—Harold Wade, Lea Harriers, L.A.C., scratch, 1; P. W. James, St. Barts., 50 yards start, 2; F. A. Waylen, L.A.C., 45, 3; A. R. Williams, Lea H., scratch, 0; sixteen others also started. Wade got up in the straight, and won by a yard; about eight separating second and third. Time, 2 min. 1-5 sec.

Long Jump.—P. R. Lowe, 18 ft. 6 in., 1; M. E. Tressider, 16 ft. 6 in., 2.

Three Miles Handicap.—H. A. Munro, scratch, 1; M. E. Tressider, 1 min. 40 sec. start, 2; P. F. Henry, 2 min. 40 sec. 3; P. Willmott, 1 min. 30 sec., 0; J. W. Eastment, 1 min. 50 sec., 0; E. Pieriot, 2 min. 50 sec., 0. Munro gradually cut down his men, and taking the lead at a little over two and a half miles, he won easily by 130 yards; ten yards separated second and third men. The winner's times were: 1 mile, 5 min. 1 sec.; two, 10 min. 14; and three miles, 15 min. 24 2-5 sec.

Sack, band, and consolation races were also on the programme. The band of the M Division of Police played selections during the afternoon, and at the conclusion the prizes were presented by Mrs. Shaw.

